# Dr. Albert Leffingwell

**Published:** 1916-10

**Authors:** 


**Dr. Albert Leffingwell,** Long Island College 1874, died at his home in Aurora, N. Y., Sept. 1, aged 75. He had retired from practice and was interested in humane work.

